# Significance of Resveratrol in Clinical Management of Chronic Diseases

**DOI:** 10.3390/molecules22081329

**Published:** 2017-08-18

**Authors:** Awais Wahab, Kuo Gao, Caixia Jia, Feilong Zhang, Guihua Tian, Ghulam Murtaza, Jianxin Chen

**Affiliations:** 1 School of Preclinical Medicine, Beijing University of Chinese Medicine, Beijing 100029, China; pharmacist5577@yahoo.com (A.W.); linfengtingchan@foxmail.com (K.G.); jcxcaixia@foxmail.com (C.J.); zhangfeilong94@gmail.com (F.Z.); rosetghdzm@126.com (G.T.); 2 Dongzhimen Hospital, Beijing University of Chinese Medicine, Beijing 100700, China; 3 Department of Pharmacy, COMSATS Institute of Information Technology, Abbottabad 22060, Pakistan; 4 Institute of Automation, Chinese Academy of Sciences, Beijing 100029, China

**Keywords:** pre-clinical studies, clinical studies, absorption, toxicity, diabetes mellitus, obesity, cardiovascular diseases, cancer, aging

## Abstract

Resveratrol could be beneficial to health and provides protection against a wide array of pathologies and age-associated problems, as evident from preclinical studies. However, a comparison of animal and human studies reveals that this dietary polyphenol cannot protect against metabolic diseases and their associated complications. The clinical outcomes are affected by many factors such as sample size. This article not only presents a comprehensive review of the current advances concerning the dose, the extent of absorption, interaction and toxicity of resveratrol in human studies, but also describes its therapeutic effects against several chronic diseases such as diabetes mellitus, obesity, cardiovascular diseases, cancer and aging and the related diseases.

## 1. Introduction

**Figure 1:**
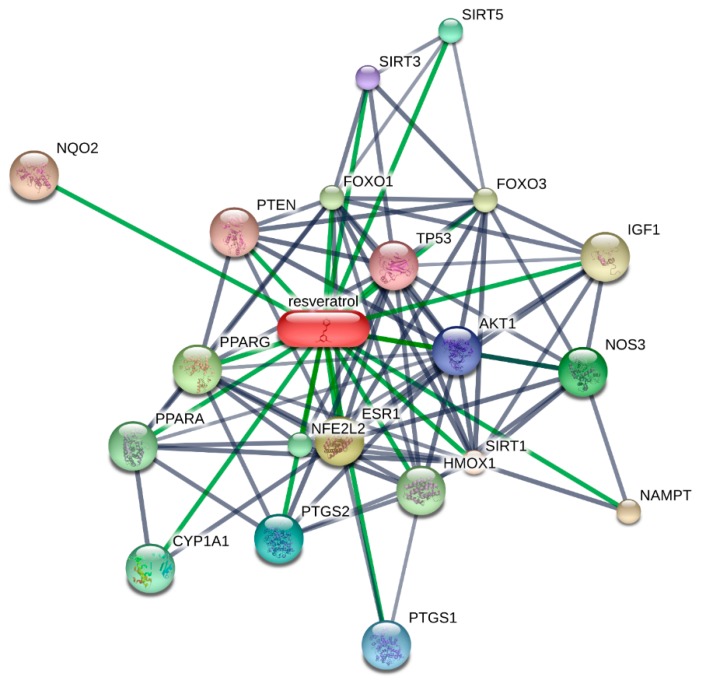
Molecular targets of resveratrol retrieved through the STITCH 5.0 database. Note: SIRT1—sirtuin 1; ESR1—estrogen receptor 1; PPARG—peroxisome proliferator—activated receptor gamma; NOS3—nitric oxide synthase 3; SIRT5—sirtuin 5; PTGS2—prostaglandin—endoperoxide synthase 2; PTGS1—prostaglandin-endoperoxide synthase 1; AKT1—v-akt murine thymoma viral oncogene homolog 1; SIRT3—sirtuin 3; TP53—tumor protein p53; PTEN—phosphatase and tensin homolog; NQO2—NAD(P)H dehydrogenase, quinone 2; NAMPT—nicotinamide phosphoribosyltransferase; IGF1—insulin-like growth factor 1; FOXO3—forkhead box O3; FOXO1—forkhead box O1; HMOX1—heme oxygenase (decycling) 1; PPARA—peroxisome proliferator-activated receptor alpha; NFE2L2—nuclear factor (erythroid-derived 2)-like 2; CYP1A1—cytochrome P450, family 1, subfamily A, polypeptide 1.

Resveratrol (3,4,5-trihydroxystilbene, RSV), found in grapes, nuts, berries, and various other plants, is a small phenolic compound. Various supplements and dietary sources contain the *cis*- and *trans*-isomers of this phenolic compound, although *trans*-RSV is the principal form found [1,2]. The pleiotropic action of RSV is responsible for its extensive use because this compound can act against various problems like energy restriction, cancer, inflammation and oxidative stress [3,4]. RSV has received a great deal of attention as a probable treatment of several human diseases since it can act on a number of molecules in the body (Figure 1).

Besides discussing the biological activities of RSV in humans, the objective of this review article is to emphasize our current understanding of RSV metabolism. Rather than emphasizing formulations containing RSV as well as other biologically active ingredients (for instance, curcumin), efforts were made to only encompass publications on the tested known quantities of RSV. Furthermore, in vitro and in vivo studies on the mode of action of RSV are also added to this review. Thus, the pleotropic effects of RSV in animals are also discussed briefly. The useful and adverse effects of supplemented RSV extrapolated to clinical findings are deliberately discussed here.

Few dose-independent adverse effects such as nephrotoxicity and gastrointestinal problems of RSV are reported also [5,6]. It is reported that 450 mg/day of RSV is a safe dose for a 60 kg person [7], but its supplementation in higher doses could be toxic. High doses of RSV (1000 mg/day or above) inhibit cytochrome P450 isoenzymes such as CYP3A4, CYP2C9 and CYP2D6 are inhibited while CYP1A2 is induced, leading to interactions with many other drugs [8]. Thus orally administered high doses of RSV indicate differences in pharmacokinetics of concomitantly administered drugs. This condition could be risky for the patients taking this supplement with comedication.

**Figure 2:**
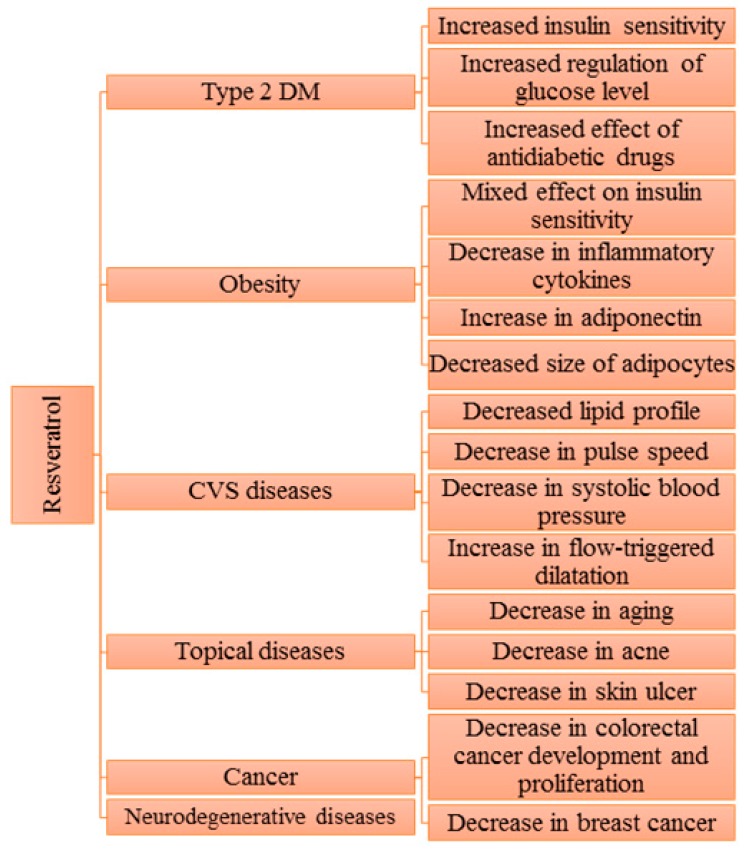
Short review of the resveratrol effects in clinical trials in patients suffering from type 2 diabetes mellitus, obesity, cardiovascular diseases (CVD) disease, skin disorders or cancer.

Figure 2 briefly summarizes the effects of RSV seen in clinical trials in patients suffering from various chronic diseases such as type 2 diabetes mellitus, obesity, cardiovascular diseases (CVD) disease, skin disorders or cancer. Findings of different treatment periods and concentrations of RSV (5 mg to 5 g) are shown here from various clinical trials. Table 1 focuses on unhealthy individuals prescribed RSV (dose, duration and route), while Table 2 describes those subjects which are healthy or do not usually take medicines such as obese individuals. From clinical trial outcomes, we observe that the least effective concentration of RSV must be set to gain maximum benefits with the least side effects.

RSV-induced longevity of different small organisms, including *Drosophila melanogaster* (fruit-fly), *Caenorhabditis elegans* (worms) and *Saccharomyces cerevisiae* (yeast), has been reported [9,10]. On the other hand, a similar RSV-triggered increase in lifespan was not observed in mice [10,11], supporting the idea that particular metabolic conditions influence the effectiveness of RSV.

An increase in insulin sensitivity in mice fed with RSV has been reported [47,48,49], while, a biphasic effect of RSV is observed in mice which are given a fat-enriched diet, causing an increase in mice weight with a low dose of RSV and vice versa [48,50].

In a study, mice were kept on physical exercise together with RSV and an increase in insulin sensitivity and number of mitochondria were observed. Moreover, an increase in the oxidation of cardiac fat and significant changes in cardiac transcription factors together with a significant decrease in blood pressure, hypertrophy and cardiac dysfunctions were observed [47,51,52]. Mechanistically, RSV-triggered amelioration of aerobic function activates SIRT1, leading to activated PGC-1 and suppressed synthesis of ROS [48].

Besides all the above effects, RSV is effective in various neuronal ailments, i.e., Huntington’s disease, diabetic neuropathy and Alzheimer’s disease [53,54]. In addition, dementia has been treated with RSV that acts on brain cells as well as metabolic disorders. Treatment with RSV caused decreased plaque formation in the brain, although no visible alteration in SIRT1 initiation or changes in amyloid-precursor protein were observed [55]. Similarly, RSV causes a decrease in hippocampal neuronal deterioration, which inhibits cognitive dysfunction and reduces the acetylation of the SIRT1 substrates (PGC-1 and p53) [56]. When rats were treated with RSV, ameliorated memory and safety from amyloid-triggered neurotoxicity via suppressed levels of lipid peroxidation and iNOS and enhanced synthesis of heme oxygenase-1 was observed [57].

RSV works efficiently against inflammation, cancer and oxidative stress, as confirmed by animal models both in vitro and in vivo methods [58]. In addition, RSV triggers the suppression of cell cycle progression and promotes the apoptosis of tumors via inhibited expression of nitric oxide synthase and restricted cancer cell growth [59] without damaging DNA [60]. RSV blocks the activity of cyclooxygenase, a vital factor of tumorigenesis [61]. Lastly, cancer cells exhibit reduced consumption of glucose under the effect of RSV, where production of reactive oxygen species (ROS) is low [62].

In spite of the ineffectiveness of RSV in neoplasia in mice [50], promising activity of RSV against cancer, particularly through SIRT1 [63], has been reported in various animal models [64,65,66]. In addition to the controversy about RSV-mediated treatment of breast cancer [67,68], studies have documented the controversial role of RSV in suppressing pancreatic cancer through up-regulation of VGEF-B and activation of apoptosis regulating factor Bax [69]. Additionally, early reports on topical use of RSV suggest that it can be used for treating skin cancer [70] and in preventing skin problems produced by UV radiation and activation of NF-kB [71].

## 2. Effect of Resveratrol on Diabetes Mellitus

The American Diabetes Association anticipates that one third of American adults will suffer from type 2 diabetes mellitus (T2DM) by 2050 [72,73]. Efficacious treatments for T2DM need to be explored to avoid its co-morbid diseases. Monkeys (*Macaca mulatta*) were kept on high sugar or fat-rich diet for two years with daily doses of 240 mg of RSV two times a day. It was reported that RSV caused significant decreases in glucose level, protection of pancreas cells and improvements in insulin actions [74,75].

The efficacy of RSV depends upon the metabolic-status of the patients, as assumed from various cellular and animal studies. RSV has a tissue-specific antihyperglycemic effect in insulin deficient states [76]. In diabetic patients, RSV causes an improvement in insulin sensitivity and glucose-regulation, but causes no effect on control subjects, as proved by a meta-analysis of 11 randomly selected clinical trials, while another meta-analysis on T2DM patients showed unaltered outcomes [77,78]. Furthermore, 5 g daily of RSV given to diabetic type-2 patients for 28 days causes a significant reduction in insulin and glucose levels in empty stomach as well as fed state [79]. Lower doses of RSV i.e., 5 mg twice a day, were given to male diabetic patients for one month and reduced level of oxidative stress markers as well as ameliorated insulin resistance were observed that could be due to activated AKT signaling [13]. As compared to administering RSV alone, when RSV was given in a dose of 250 mg daily in combination with an antihyperglycemic agent for a trial period of three months, an additive effect on the glucose lowering activity of RSV was noted, besides glycated hemoglobin A_1c_ (HbA_1c_), reduction of systolic blood pressure and total cholesterol. A similar study showed unaltered findings except HbA_1c_ after 6 months [12,80]. Another study on concomitant administration of RSV and metformin showed that combined therapy may ameliorate the curative effect of diabetic disorders and the associated complications [81]. Correspondingly, 1 g daily dosing of RSV for 45 days in diabetic patients receiving anti-diabetic treatment, not only provided typical anti-diabetic effects, but it also provided an excellent protection against type-2 diabetes [82]. Despite these observations, when five T2DM patients were kept on a combination therapy of RSV (0.5–3 g/day) and hypoglycemic agents for 3 months no decrease in glucose metabolic markers and HbA_1c_ level was observed [83].

In monkeys, RSV causes dose-dependent harmful effects on pancreas development during pregnancy and leads to pancreatic islet hypervascularization, a possible cause of tumors in *M. mulatta* babies [84,85]. Contrasting results were found in another study, i.e., RSV administered to pregnant patients having T2DM averts embryonic malformation in diabetic-rat dams. This effect could be mediated through normalization of elevated blood sugar levels and enhanced oxidative stress [86]. Therefore, keeping in mind both the adverse and beneficial effects of RSV, a lot of care should be taken while prescribing it during pregnancy.

## 3. Effect of Resveratrol on Obesity and the Related Problems

The prevalence of obesity presents a serious health and societal issue all over the world. Type-2 diabetes, hypertension and dyslipidemia are various metabolic issues that cause accumulation of fat in the body. This further leads to cardiac diseases and shortening of life. Changes in the structure of the myocardium and endothelium arise because of excessive fat accumulation. In addition, inflammation and thrombosis occur due to the secretion of many adipokines [87]. Many reports explain that RSV causes change in cardiac health such as inhibition of plaque development, platelet accumulation, lipid metabolism, endothelial role, inflammation and stress markers [88,89,90].

Insulin resistance and T2DM are often associated with obesity. Studies in obese patients treated with RSV have shown some inconsistent findings, i.e., while some studies showed an improvement in insulin sensitivity, no such outcome were observed in some other studies [19,20]. These differences could be because of dissimilar sample size and study objectives, including dose, dosage form and route and duration of administration.

One of the main causes of CVD is arteriosclerosis in which there is decrease in the quantity of high-density lipoprotein (HDL) and an increase in low-density lipoprotein (LDL) [91]. In this regard, another study assessed the advantages of RSV intake on lipid profile and showed non-significant changes in lipid parameters such as triglycerides, cholesterol, LDL and HDL, while this nominal change did not depend on dose, study duration, the CVD risks [91]. It is clear from this observation that the RSV effect against CVD could not be caused by triglyceride levels. It is documented that using 250 mg/day of RSV for three months significantly decreases cholesterol, LDL, and ApoB in cardiac and diabetes type 2 patients [12,24,92].

CVD is also caused by hypertension in addition to dyslipidemia [93]. A current analysis has revealed that systolic blood pressure decreases when treated with RSV at a dose of ≥150 mg/day having no effect on diastolic blood pressure. A recent meta-analysis showed that treatment with ≥150 mg/day of RSV decreases systolic blood pressure without affecting diastolic blood pressure [12,82,94]. There is an additional significant biomarker of CVD which is flow-mediated dilation of the brachial artery that has a direct impact on hypertension. Considerable RSV-triggered rise in flow mediated dilation is observed in all post-myocardial infarction patients, post-menopausal women and obese men that were not treated for their borderline high blood pressure [17,26]. It is proposed that RSV causes an improvement of endothelial function by stimulating Ca^+2^-activated potassium-channels and increasing nitric oxide signaling [26].

A chronic, low-grade inflammation triggered by a bulk of cellular nutrients lying in metabolically active tissues is known as obesity-induced meta-inflammation. Vascular dysfunction and obesity are promoted by high levels of inflammatory markers [95]. Key sources of ROS in the obese people are fat deposits, which harm, on release into the blood, various organs and tissues [96]. Obesity-related comorbidities can be treated with RSV. In a previous study, rhesus monkeys were nourished with high calorie diets and RSV for 24 months resulting in decreased adipocyte size; this outcome is also verified in a recent study on humans [33,75]. The steady state of mRNA levels of numerous inflammatory markers including IL-1 and IL-6 as well as diet-induced NF-kB activation are also reduced by RSV [75]. In various clinical studies, similar effects of RSV on plasma pro-inflammatory cytokine levels are described. In CVD patients supplemented with RSV (350 mg/day) for six months to one year, there was a reduced production of IL-6, IL-10 and TNF [14,97]. Treatment with RSV increased various anti-inflammatory biomarkers such as adiponectin while it decreased inflammatory biomarkers such as high sensitivity CRP (hs-CRP) [24]. Non-alcoholic fatty liver disease (NAFLD) patients were treated with RSV 500 mg for 3 months and a decrease in hepatocellular apoptosis and inflammatory markers were noted, while some studies reported that there was non-significant effect of RSV on cytokines in NAFLD patients [20,26]; thus the anti-inflammatory activity of RSV is still being debated. As discussed above, the anti-inflammatory activity of RSV is affected by the patient’s metabolic condition [14,97]. Various studies have demonstrated a suppression in oxidative stress markers in patients having metabolic syndrome treated with RSV [36], while in non-obese and healthy subjects, no important change in CRP and LDLox levels occurs [98]. Central arterial wall stiffness is caused by a chronic inflammatory milieu, which further leads to CVD. Various reports show that aortic stiffness can be analyzed by pulse wave velocity (PWV) [99], but still there is no successful treatment to decrease it. In a recent study, a non-significant increase in PWV is reported when RSV was given to non-human primates kept on high fatty sugar regime at a concentration of 240 mg two times a day for 24 months [100]. RSV therapy also causes reduction in caspase-3 activity and levels of 4-hydroxynonenal (a lipid peroxidase marker). These findings are supported by a defense mechanism of RSV against oxidative stress and apoptosis [100]. In addition, studies were also carried out in patients with metabolic syndrome and treated with RSV. As a result, lowering of their CVD marker levels as well as reduced obesity was noted.

## 4. Effect of Resveratrol on Neurodegradation

Neurodegenerative diseases are those diseases which are characterized by inflammatory conditions, along with chronic and progressive pathologies, whereas activation of microglia causes neuronal damage in the central nervous system (CNS) and increased ROS generation. RSV can cause resistance against neurodegeneration and preserve cognitive functions when given in combination with flavonoids, as proved by various trials and epidemiological reports [101,102,103].

To observe changes in cognitive function by treatment with RSV, a few clinical trials have been carried out in healthy patients only. It has been suggested that cerebral arterial vasodilation should be increased by enhancing systemic vasodilator function to increase cognitive performance. It was observed that oxygen extraction and cerebral blood flow was increased in a dose dependent manner in healthy men after short-term treatment with RSV (after a 45-min resting absorption period of 250 or 500 mg of RSV), while it had no marked effect on their cognitive function [39]. A significant reduction in fatigue was observed when 500 mg of RSV was given during 28-day diet supplementation, but there was no significant effect on chronic cerebral blood flow, health status or sleep patterns.

In order to increase RSV access to the brain and to overcome the RSV bioavailability problem, the formulation was changed by the addition of piperine. When RSV and piperine were given in combination, there was increased cerebral blood flow compared to RSV alone or placebo and there was no effect on blood pressure, cognitive function and mood [41]. It was observed that RSV supplementation neither caused any important change in concentration and cognitive function, nor did subjects show any improvement in flow-mediated dilation. From these observations, it was concluded that participants having cognitive impairments could be treated with RSV supplementation because it might be only effective in cognitive impairments [40]. After a 26-week supplementation with RSV and quercetin in healthy overweight older adults, the results showed an enhancement in hippocampal functional connectivity along with increased metabolism of glucose in the brain and also improved memory performance [42]. Concerning the reduction of brain inflammation by RSV, there is very limited information. Another group of researchers used RSV to treat subjects with mild to moderate Alzheimer’s disease for 52 weeks and found that RSV activates SIRT1, decreases neuroinflammation, suppresses cerebrospinal fluid metalloproteinases and triggers adaptive immunity [104]. Moreover, RSV prevented neurodegeneration of dopaminergic neurons of model mice triggered by rotenone via reduction in nigral iron levels [105]. Similar observations were noted in mice with multiple sclerosis, where RSV supplementation improved the disease condition via maintenance of blood brain barrier integrity [106].

## 5. Effect of Resveratrol on Aging and Topical Diseases

Researchers all over the world are investigating the process of aging and its accompanying pathologies, in an effort to discover the mythological fountain of youth. To treat and prevent various diseases linked to age, recent knowledge has helped us much. In pathologies related to age such as neurodegenerative diseases, CVD, cancer and T2DM, treatment with RSV possesses marked useful effects, however no studies have been carried out to check the impact of RSV on longevity in primates, as well as in humans. It is remarkable that RSV triggers identical modifications in gene expression arrays as a calorie restriction mimetic [107]. In nonhuman primates, the effect of calorie restriction mimetic on lifespan has been reported to be controversial [108,109], which could be due to hereditary factors of the monkeys and diet composition.

Even though there is limited studies on humans, when RSV is applied topically on human skin, it decreases the formation of sunburnt cells and protects the skin from the harmful effects of sun-rays [110]. There was improvement in depth of wrinkles and an amelioration of skin roughness, skin elasticity and its moistness along with a decrease in age-spot color intensity [111]. In human skin, particular receptor sites for RSV are present. This suggests that this polyphenol might be effective in skin disorders related to aging [112]. When cyclodextrin excipient was given in combination with RSV, the signs of aging were improved [113]. When RSV was given to volunteers with acne vulgaris, it showed anti-acneic properties [114]. In T2DM patients, foot ulcer size was reduced when RSV treatment was given for 60 days (50 mg/twice a day) [115]. A promising treatment outcome was achieved when topical formulation of RSV was applied to the chemically pealed rat skin. It results in thicker dermis and epidermis that indicates greater skin vitality, probably due to greater collagen production. In enhances skin elasticity and firmness leading to reduced skin wrinkles and aging [116].

## 6. Effect of Resveratrol on Cancer

For assessment of upregulated Wnt signaling, a hazardous element for causing cancer of colon [7], pure RSV in a dose of 20/80 mg or dried grape powder in a dose of 80/120 g/day was administered to eight humans with colorectal cancer [9]. RSV had no effect on cancerous mucosa, however it showed a potential role of RSV in preventing mucosal cancer. To get rid of a limitation of this study, namely the small sample size, an additional 20 colorectal cancer patients were treated with a micronized form of RSV with a dosage regimen of 0.5 g/1 g and evaluated for 8 days before surgery: a reduction of 5% in the rate of cellular proliferation in colorectal cancer tissue with no change in histopathology in pre- and post-surgical tissues was noted [22]. Furthermore, by concomitant use of SRT501 in six patients suffering from colorectal cancer metastasized to the liver, an increased bioavailability of RSV as well as enhanced stimulation of the apoptotic indicator caspase 3 was noted [5]. Many side effects have been observed with the use of 5 g/day of SRT501 in patients with refractory or relapsed myeloma and one patient died as well during treatment [117]. Decreased methylation of the tumor suppressor gene RASSF1 was observed in patients with breast cancer taking RSV for 3 months (5 or 50 mg in two doses/day) leading to reduced levels of prostaglandin E_2_ (PGE2) which promotes cancer [23]. A preclinical study reported an improvement in the autoimmune system through RSV-mediated increase in T-cells [118]. In addition, the antitumor potential of RSV and its mode of action in cisplatin-resistant human oral cancer CAR cells was tested and excellent autophagic and apoptotic activity were found [119]. An in vivo study described RSV supplementation to U87 glioma flank xenografted mice. The results illustrated a promising potential of RSV against the induced mutagenicity, most probably due to AKT inactivation [120]. Moreover, a clinical study has described the human safety of pulverized muscadine grape skin (PMGK), which enhances prostate-specific antigen doubling time in non-metastatic, biochemically recurrent prostate cancer patients. PMGK contains RSV in appreciable concentration [121]. Although the abovementioned results are quite amazing, we still have to understand them carefully because of the small sample size and weak relationships. Despite these highly health beneficial results, more studies on RSV are still required to confirm whether RSV is the best choice to treat cancer.

## 7. Effect of Resveratrol on Exercise-Induced Outcomes

For the improvement of health, prevention of T2DM, the ameliorated vascular function and reduction of cardiovascular risks, exercise is an effective tool. In older age, various health outcomes related to aging process can be minimized by aerobic exercise [122]. In obese subjects, SIRT1 and PGC-1 protein levels were increased and skeletal muscle AMPK was activated after supplementing RSV-rich diet, but not in healthy individuals and T2DM patients [29,83]. The effect of exercise was positive on the inflammatory and skeletal muscle metabolic status in aged men but it was annulled by RSV [123]. It showed consistency with a report that claimed a negative impact of antioxidant supplementation through oral route on exercise in older inactive adults [123]. Another study was carried out on 16 young men who were initially given a dose of RSV daily and then before and after a four-week sprint-interval training program. The results revealed that participants treated with RSV despite of having maximum uptake of oxygen alike the placebo group showed a smaller enhancement in the ability of fat burning during exercise and anaerobic conditions [28,124,125].

Athletes receiving exercise training along with 150 mg of RSV per day did not experience any increase in the normal training response triggered by high intensity training and low dose of RSV [28]. In addition, athletes conducted exercise training with concomitant use of RSV and quercetin experienced substantial decline in exercise-triggered lipid peroxidation with no alteration in inflammation [126]. In elderly men, RSV impeded the positive outcomes of a 48 days exercise training on cardiovascular status [127]. With exercise training either using RSV supplementation or not, no change was observed in SIRT1 protein expression. Neither the activity of oxidative proteins and muscle endurance was significantly improved by the use of RSV, not it showed any effect on decrease in protein carbonylation level and skeletal muscle TNF mRNA content which are enhanced by doing exercise [127].

## 8. Effect of Resveratrol on Healthy Subjects

As mentioned previously, the metabolic condition of patient seems to state the effectiveness of RSV treatment. Clinical trials of RSV treatment, when performed in the patients having insulin resistance due to obesity, diabetes, cancer or cardiovascular disease, confirmed positive results, but a negative effect was observed in people in good health [6,30]. In these clinical trials, the majority of the people were obese, however they were otherwise healthy and not getting any medications for cardiovascular, metabolic or inflammatory disease. In healthy subjects, positive responses with RSV treatment, increases in triglyceride level of plasma [29,37], response suppression of postprandial glucagon [32], improved metabolic mobility with lesser HOMA-IR index [29], and decreased level of cytokines [29,34,35] were stated. RSV not only reduces resting metabolic rate, but also ameliorates the respiratory system in the overweight healthy people [29]. As compared to treatment with RSV only [30], a significantly higher decline in the resting metabolic rate was noted when healthy participants were supplemented with RSV diet and another polyphenol, epigallocatechin gallate [31]. It is evident from these findings that the effects of RSV are more prominent in diseased subjects than in healthy ones [128].

## 9. Supplementation of Resveratrol and Future Perspectives

There are adequate logical facts to deem that RSV is a compound of immense importance for human health. To recommend RSV to be useful for human health, several aspects still require interpretation as discussed below:

### 9.1. Mechanism of Action of RSV

There are still controversies regarding the mechanism of action of RSV due to nonspecific RSV targets [129]. Previous studies showed that RSV directly activates SIRT1 [130], however, an indirect activation of SIRT1 through inhibition of cAMP phosphodiesterases by RSV was described in a latest study [131]. However, suggestions show that RSV directly triggers SIRT1 as well as through indirect mediators [132,133], while RSV affects metabolism through SIRT1-mediated AMPK activation [47,134]. Health outcomes of RSV could be better understood if its cellular targets were identified and validated [135]. Figure 1 summarizes the reported molecular targets of RSV, as retrieved from STITCH, which is a database rich in cellular targets of natural compounds.

### 9.2. Dose and Dosage of RSV 

RSV levels in the diet are too low to obtain beneficial health effects [47,136]. In addition, RSV bioavailability is affected by race, sex, age, gut microbiota [46], diet, physical exercise, genetic polymorphism [137] and differences in gene expression between individuals. Thus, it is mandatory to determine the dose, dosage form, frequency and tool of delivery of RSV. Moreover, RSV is quickly metabolized in the alimentary canal, thus new drug delivery tools are required to deliver it directly to the blood or the target site for improved therapeutics [138]. In most current studies, RSV has been administered as a conventional powder or encapsulated grains or as a solution, solid lipid nanoparticles [139,140] and topical dendrimers [141].

### 9.3. Side Effects and Interaction of RSV

Animals can tolerate RSV in small doses, however high doses of RSV show extensive side effects [117,142]. However, further studies are required for the evaluation of supplementation effect of RSV on human health and discover the methods for treatment and prevention of its side effects. Additionally, it is important to understand the interactions of RSV with drugs and other supplements to diminish undesirable happenings [143]. Since cytochrome P450 enzymes are modulated by RSV, it can detoxify and metabolize drugs and xenobiotics in the liver [144]. Thus, future experiments should be focused on the effects of RSV after concomitant administration with other drugs, xenobiotics and diets. Actually, the improvement in RSV bioavailability is reported when RSV is combined with other supplements [137,145,146].

## 10. Conclusions

Resveratrol could be useful to protect health against a number of pathologies and ageing problems, however, the comparative evaluation of animal and human studies shows that RSV cannot protect against metabolic diseases and their relevant complications. The clinical findings are influenced by many factors such as sample size and study objectives. Till now, small sample size and high dosage levels were used to conduct most clinical trials to assess the significance of RSV in chronic diseases. Consequently, it is not easy to determine exact safety range and therapeutic effectiveness of specific RSV doses for specific populations. Before prescribing RSV, the patients should be advised properly for effective treatment with minimum side effects. Further evaluations are needed before declaring RSV as a beneficial health compound for humans.

## Figures and Tables

**Table 1:** Clinical findings on diseased participants treated with resveratrol.

Disease	No. of Treated Subjects (Males, Females or Both)	Dose of Resveratrol	Duration of Treatment in Days	Important Findings	Reference
T2DM	57 (Both)	250 mg	120	Ameliorated profiles of lipids (total cholesterol by 7.8%), total proteins (by 4.36%) and hemoglobin A1c (by 3.4%)	[12]
T2DM	19 (Males)	5 mg	30	Reduced blood glucose levels and ameliorated insulin resistance	[13]
T2DM	35 (Males)	8 mg	365	Down-regulation of various cytokines including CCL3 and TNF	[14]
Obesity	8 (Both)	1000–2000 mg	14	Non-significant effect on lipid profile but lowered synthesis of apoB-48 and apoB-100	[15]
Obesity	74 (Males)	500 mg	105	Increased bone density	[16]
Obesity	19 (Both)	30, 90 or 270 mg	21	Significantly elevated FMD	[17]
Impaired glucose tolerance (Obesity)	10 (Both)	1000–2000 mg	28	Ameliorated insulin sensitivity	[18]
Metabolic syndrome (obesity)	24 (Both)	500 mg	90	Significantly reduced weight, BMI, fat mass, and weight, Significant effect on insulin sensitivity.	[19]
Non-alcoholic fatty liver disease	20 (Males)	3000 mg	56	Non-significant effect on insulin function and fat distribution	[20]
Non-alcoholic fatty liver disease	49 (Both)	500 mg	84	Significant decline in hepatic inflammatory markers	[21]
Colorectal cancer and hepatic metastases	9 (Both)	5000 g	21	Excellent distribution of RSV in body	[5]
Colorectal cancer	20 (Both)	500 mg	8	Reduced proliferation in in cancerous tissue	[22]
Colon cancer	8 (Both)	80 mg	14	No effect on Wnt signaling in cancerous mucosa	[9]
Breast cancer	31 (Females)	5 or 50 mg	84	Modified methylation of RASSF-1 (an indicator of breast cancer)	[23]
Angina pectoris	116 (Both)	20 mg	60	Significant decline in hs-CRP	[24]
Coronary heart disease	75 (Both)	8 mg	365	Elevated levels of adiponectin and decrease in PAI-1	[25]
Myocardial infarction	40 (Both)	10 mg	120	Ameliorated functioning of endothelium and left ventricle as well as reduced level of LDL	[26]

**Table 2:** Clinical investigations on healthy subjects (participants receiving no other medicine) supplemented with resveratrol.

Aim (To Study the Effect of Resveratrol)	No. of Treated Subjects (Males, Females or Both)	Dose of Resveratrol	Duration of Treatment in Days	Important Findings	Reference
On metabolic profile	32 (Both)	300 or 1000 mg	84	Suppressed levels of fasting glucose (by 1.67 ± 1.51 mg/dL at 300 mg dose) and bilirubin	[27]
On metabolic profile	24 (Males)	500 mg	28	Non-significant change in the markers of obesity	[6]
On metabolic profile	16 (Males)	150 mg	28	Non-significant effect on aerobic or anaerobic capacity	[28]
On metabolic profile	11 (Males)	150 mg	30	Enhanced oxidative phosphorylation and reduced postprandial energy expenditure and adipose tissue lipolysis	[29]
On metabolic profile and insulin sensitivity	29 (Females)	75 mg	84	Non-significant effect on metabolic rate or the insulin sensitivity	[30]
On energy expenditure and substrate metabolism	18 (Both)	200 mg	3	Significantly improved fasting and postprandial energy expenditure	[31]
On postprandial incretin hormone levels	10 (Males)	150 mg	30	Significant suppression of postprandial glucagon response	[32]
On adipose tissue morphology	11 (Males)	150 mg	30	Significantly reduced adipocyte size as well as ameliorated insulin sensitivity	[33]
On markers of oxidative and inflammatory stress.	20 (Both)	40 mg	42	Diminished levels of oxidative stress and inflammation biomarkers	[34]
On markers of oxidative and inflammatory stress.	10 (Both)	100 mg	Single dose	Suppressed the increase in oxidative stress, lipopolysaccharide and LBP concentrations	[35]
On markers of oxidative stress in obese patients.	32 (Both)	150 mg	Single dose	Significantly higher antioxidant effect of RSV triphosphate (RTP) and grape extract than RSV	[36]
On inflammation and oxidative stress markers in smokers.	50 (Both)	500 mg	90	Diminished levels of C-reactive protein and triglycerides, and increased total antioxidant levels	[37]
On human mononuclear cells upon bacterial stimulation.	10 (Males)	5000 mg	Single dose	Increase in TNF-levels while IL-10 levels were decreased.	[38]
On cerebral blood flow and cognitive performance	9 (Males)	250 mg and 500 mg	Single dose	Increased cerebral blood flow	[39]
On flow-mediated dilation (FMD) and cognitive performance.	28 (Both)	75 mg	42	No adverse side effects	[40]
On flow-mediated dilation (FMD) and cognitive performance	6 (Males)	250 mg	Single dose	Significant increase in FMD	[41]
On cognitive performance	46 (Both)	200 mg	182	Significantly improved memory retention	[42]
On endothelial response and vascular markers.	41 (Both)	400 mg	30	Protection against atherosclerosis	[43]
On systemic sex hormone levels	40 (Females)	500 mg	84	Significant increase in sex-hormone binding globulin	[44]
Of administered form on bioavailability	15 (Both)	40 mg	Single dose	Ameliorated absorption in dry powder form	[45]
Of gut microbiota on metabolism of resveratrol	22 (Both)	0.5 mg/kg	Single dose	Variable metabolism of resveratrol among individuals	[46]
